# Psychiatric and Behavioral Complications of GPi DBS in an Adolescent with Myoclonus Dystonia

**DOI:** 10.1155/2019/1947962

**Published:** 2019-06-04

**Authors:** Graciela Kriegel, Melanie I. Stuckey

**Affiliations:** ^1^Ontario Shores Centre for Mental Health Sciences, Whitby, Canada; ^2^University of Toronto, Department of Psychiatry, Toronto, Canada; ^3^University of Ontario Institute of Technology, Faculty of Health Sciences, Oshawa, Canada

## Abstract

Myoclonus dystonia is a rare movement disorder that often causes significant disability. Deep brain stimulation of the internal pallidum (GPi DBS) is a recommended treatment for those who do not respond to pharmacotherapy or who have intolerable side effects. This paper reports on the case of a 17-year-old male who was admitted to a tertiary level mental healthcare facility for treatment of psychiatric and behavioral symptoms thought to be related to GPi DBS. Prior to GPi DBS insertion, the patient was diagnosed with anxiety and mild obsessive compulsive disorder (OCD). Following insertion, his OCD became severe and he developed depression, Tourette syndrome, and stuttering. His first admission to a psychiatric unit was for management of a manic episode following treatment for depression with fluoxetine, and he began to exhibit severe aggressive behavior. GPi DBS was turned off, but there were neither changes in dystonic movements nor improvement in aggressive behavior or psychiatric symptoms, though stuttering improved. The patient was transferred to a secure treatment centre where he was able to gain control over his behaviors with intense dialectical behavior therapy, but the aggressive behavior and safety concerns continue to persist today.

## 1. Introduction

Myoclonus dystonia syndrome (MD) is a rare movement disorder that typically begins in childhood and adolescence and often causes disability. MD has an autosomal dominant inheritance pattern mostly due to a pathogenic mutation in the *Ɛ*-sarcoglycan gene (SGCE gene) on chromosome 7q21 (also known as DYT11), which can be identified in 30 to 50% of patients. At least one other locus (DYT15, 18p11) has been linked to the disease [1]. It has been linked to an increased prevalence of psychiatric disorders including major depression [2], obsessive compulsive disorder (OCD), and substance abuse [3].

MD symptoms are generally managed with pharmacotherapy. For those who do not respond to pharmacotherapy or who have intolerable side effects, deep brain stimulation of the internal pallidum (GPi DBS) has been recommended as an alternative treatment option; however, GPi DBS may exacerbate psychiatric symptoms [4]. While there is not a robust body of evidence due to the rarity of the disease, the literature suggests that GPi DBS can have adverse effects on mood and behavior. Previous studies have shown a potential link between stimulation of the subthalamic nucleus [5] and the globus pallidus [6] with DBS and the development of mania, particularly in the treatment of Parkinson Disease. Violence and aggression have also been linked to GPi DBS: a case was reported of an adolescent who developed severe aggressive behavior after GPi DBS insertion to manage Juvenile Onset Parkinson Disease [7].

This paper presents the case of a 17-year-old male diagnosed with MD and treated with GPi DBS, who developed mania and severe aggressive behavior following a change in frequency of GPi DBS stimulation.

## 2. Case Report

**Figure 1:**
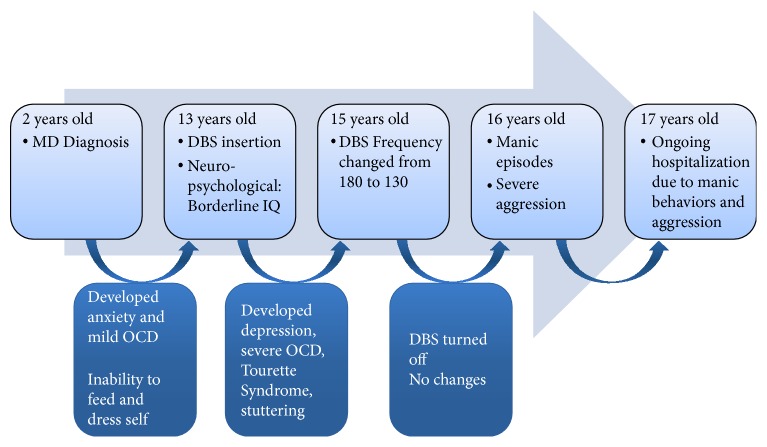
Timeline of progression of myoclonus dystonia, psychiatric, and behavioral complications.

The patient was diagnosed with MD at the age of two years old. He had a strong family history of MD and his family tested positive to DYT15. Although he was symptomatic from an early age, he tested negative to DYT15. His MD symptoms worsened to the point that he required assistance for activities of daily living, such as feeding and dressing. His community doctors attempted several medications for treatment, all of which were poorly tolerated by the patient. At the recommendation of his neurosurgeon, GPi DBS was inserted for treatment at the age of 13 years old. Stimulation was initiated at a frequency of 180 Hz. Notably, people of the DYT15 kindred who tested negative may achieve fewer benefits from DBS compared to those who tested positive [8]. Assessment of MD symptoms 18 weeks after surgery indicated 70% improvement (Figure 1).

Prior to surgery, the patient presented with anxiety and mild OCD symptoms. Approximately one year after GPi DBS surgery, the frequency of stimulation was decreased to 130 Hz. Soon after this change, the patient's mother noticed a change in his mental state and the patient developed depression, severe OCD, Tourette syndrome, and stuttering. Despite no family history of mood disorder, the patient developed mania following treatment for symptoms of depression with fluoxetine and required admission to a psychiatric unit at the age of 16 years old for management. The patient, who had presented with anxiety and mild OCD symptoms prior to surgery, began to exhibit severe aggressive behavior.

We first assessed the patient at the age of 17 years old when he was admitted to our tertiary level adolescent psychiatric unit after displaying severe aggressive behavior and homicidal ideation by threatening his mother with a knife and chasing his father with a butcher's knife. During his admission he had two manic episodes that were not triggered by the use of antidepressant medication. More than 45 incidents of unprovoked aggression were reported, including threats to kill and rape staff, insulting, lashing out at staff, spitting, kicking, punching, and destroying property. The aggression was at times an impulsive reaction, but other times it was premeditated aggression accompanied by inappropriate smiling and expressions of feelings of pleasure from hurting others. Multiple staff injuries resulted from these behaviors. In one instance, the aggression escalated such that a Special Weapons and Tactics (SWAT) team was called to manage a highly dangerous situation. The patient was placed in seclusion, where he remained for several weeks, as attempts to discontinue the seclusion were unsuccessful. Notably, during these aggressive incidents/episodes, reductions in dystonic movements and improvements in stuttering and the ability to express himself were observed.

Medication tried included Seroquel XR 200 mg, which was switched to Olanzapine up to 20 mg. Valproic acid was added up to 750 mg (low doses were administered as recommended by the neurology team) but no improvements in behaviors or symptoms were seen.

When the GPi DBS was turned off, the previous improvements in dystonic movements were maintained; the patient maintained independence in feeding and dressing while stuttering improved. Manic symptoms were well-controlled but violent behavior did not remit.

Due to the ongoing difficulties in management on the unit, the patient was transferred to a highly secure treatment centre, where he was able to gain significant control over his behaviors through intense dialectical behavior therapy, but the aggressive behavior and safety concerns continue to persist up to the present time. The GPi DBS was turned on for a few more months and turned off again due to increased stuttering, with no further improvement.

## 3. Discussion

Literature examining the psychiatric and behavioral side effects of GPi DBS in adolescents with MD is lacking. Mania has been reported in 0.9-1.7% of individuals treated with DBS [5], and in one case report, a 40-year-old man developed manic symptoms after the frequency of GPi DBS was reduced [6]. Our patient also developed manic symptoms following reduced frequency of GPi DBS.

In the case presented, our patient began to exhibit aggressive behaviors, which escalated to violence. Similarly, an adolescent treated with GPi DBS for Juvenile Onset Parkinson Disease showed increased mood lability, agitation, and homicidal gestures [7], a side effect also reported by Borggraefe et al. [9] in one child, following bilateral pallidal stimulation with a delay of 36 months after implantation. Even though we are still lacking data from pediatric populations, Burdick et al. showed significantly higher anger scores after DBS, in both STN and GPi groups, suggesting that it may be a lesional effect [10]. There is a growing number of cases reporting behavioral and psychiatric side effects that suggest, in some cases, irreversible symptoms. Further research is strongly recommended in anticipation of future use of DBS to treat a number of neurological and psychiatric conditions, especially in the pediatric population.

## 4. Conclusion

Concerns and questions about the use of pediatric GPi DBS have been raised by many experts, especially when considered for the treatment of several psychiatric illnesses. Even though it is difficult to differentiate the psychiatric symptoms that could be related to the previous neurological condition from neurological damage caused by the GPi DBS or to a comorbid psychiatric condition [11], in the case of this patient, the temporal relationship between the change in frequency of DBS stimulation and presentation of mania and severe aggressive behavior may suggest some association. Larger scale studies are needed to determine causality.
